# Post-anesthetic CPS and EQUUS-FAP scores in surgical and non-surgical equine patients: an observational study

**DOI:** 10.3389/fpain.2023.1217034

**Published:** 2023-07-12

**Authors:** Rachel Anne Reed, Anna M. Krikorian, Rose M. Reynolds, Brittany T. Holmes, Megan M. Branning, Margaret B. Lemons, Michele Barletta, Jane E. Quandt, Charlotte C. Burns, Stephanie C. Dantino, Daniel M. Sakai

**Affiliations:** ^1^ Department of Large Animal Medicine, College of Veterinary Medicine, University of Georgia, Athens, GA, United States; ^2^ Department of Small Animal Medicine and Surgery, College of Veterinary Medicine, University of Georgia, Athens, GA, United States

**Keywords:** anesthesia, analgesia, horse, pain score, pain

## Abstract

**Background:**

Equine pain scoring may be affected by the residual effect of anesthetic drugs.

**Objectives:**

To compare pain scores in the hours immediately following anesthetic recovery to baseline pre-anesthetic scores in equine patients undergoing surgical and non-surgical procedures.

**Study design:**

Clinical observational study.

**Methods:**

Fifty adult horses undergoing anesthesia for surgical or non-surgical procedures were enrolled. Horses underwent pain scoring using the Composite Pain Score (CPS) and Equine Utrecht University Scale for Facial Assessment of Pain (EQUUS-FAP) prior to anesthesia (T0) and following anesthetic recovery to standing, every hour for 5 h (T1-T5). Data were analyzed using a generalized linear mixed effects model. A post-hoc Dunnett's test for multiple comparisons was performed for variables where an effect was detected.

**Results:**

Mean (95% confidence interval) CPS scores for T0-T5 were 1.6 (1.2–2.0), 6.8 (6.0–7.6), 5.1 (4.3–5.9), 4.3 (3.4–5.2), 3.7 (2.8–4.6), and 2.8 (2.0–3.6) and EQUUS-FAP scores were 0.6 (0.3–0.9), 3.0 (2.5–3.5), 1.9 (1.6–2.2), 1.1 (0.8–1.4), 0.6 (0.4–0.8), and 0.7 (0.4–1.0), respectively. For the CPS, scores greater than 5, and for the EQUUS-FAP scores greater than 3, are consistent with minor pain. There was no effect of type of procedure (surgical vs non-surgical) on CPS or EQUUS-FAP scores. There was an effect of time with CPS scores significantly greater than baseline at T1-T5 and EQUUS-FAP scores significantly greater than baseline at T1 and T2.

**Main limitations:**

Discomfort caused by hoisting was not quantified and it was difficult to ascertain if this affected the results.

**Conclusions:**

Post-anesthetic pain scores may be influenced by the residual effect of anesthetic agents for as long as 5 h and 2 h for the CPS and EQUUS-FAP, respectively.

## 1. Introduction

Pain scoring is an important component of providing adequate analgesia to equine patients and is one of the clinical recommendations for primary practice made by the British Equine Veterinary Association Analgesia Panel (1). In recent years, several equine pain scoring systems have been investigated and validated for different types of pain. These scoring systems have been reviewed elsewhere (2, 3).

The Equine Composite Pain Score (CPS) and the Equine Utrecht University Scale for Facial Assessment of Pain (EQUUS-FAP) are two systems that have been validated for scoring of orthopedic pain and visceral pain (4–8). However, it is unclear how the residual effects of general anesthesia may affect the accuracy of these pain scoring systems in the hours immediately following anesthetic recovery. Indeed, the effect of general anesthesia on the equine stress response has been well documented (9–11) and it can influence factors assessed in pain scoring systems. The residual effects of general anesthesia on physiologic variables including heart rate, respiratory rate, body temperature, and borborygmi included in the CPS could falsely alter the resulting pain score. Additionally, the effect of anesthesia on patient position, stance, and appearance could falsely increase both CPS and EQUUS-FAP pain scores resulting in unnecessary rescue analgesia and associated systemic adverse effects.

The objective of the study presented here was to determine the effect of general anesthesia on CPS and EQUUS-FAP scores in the hours immediately following recovery in horses undergoing surgical and non-surgical anesthetic episodes. It was hypothesized that pain scores would be significantly higher than baseline immediately following anesthetic recovery regardless of whether the patient underwent a surgical or non-surgical procedure.

## 2. Materials and methods

### 2.1. Study design

A prospective observational study of horses presenting to the University of Georgia Veterinary Teaching Hospital for elective anesthesia was performed between May 2022 and August 2022. Ethical approval from the University of Georgia Clinical Research Committee was waived prior to the start of data collection as the study was purely observational and no horse would receive any unique treatment as a result of the study. Inclusion criteria comprised: healthy adult equine patients presenting for elective anesthesia for surgical or non-surgical procedures. Exclusion criteria comprised: behavioral attributes making it unsafe to perform scoring and scheduled time of anesthesia when no investigators would be available for scoring.

Horses were housed in 3.7 × 3.7 m stalls where they acclimated to the hospital for 12–24 h prior to anesthesia. Horses were fed hay and grain thrice daily with the exception of the morning prior to anesthesia when they received a small flake of hay only. Water was made available at all times.

### 2.2. Anesthetic events

All horses were anesthetized utilizing a similar anesthetic protocol. Subjects received a pre-anesthetic non-steroidal anti-inflammatory drug (NSAID), either phenylbutazone (2.2 mg/kg; Phenylbutazone; Covetrus, Portland, ME, USA) or flunixin meglumine (1.1 mg/kg; Banamine; Merck Animal Health, Rahway, NJ, USA) IV, based on the clinician's preference, and they were sedated with intravenous (IV) xylazine (1.1 mg/kg; AnaSed; Akorn Animal Health, Gurnee, IL, USA). Immediately prior to induction of anesthesia, horses received hydromorphone (0.04 mg/kg; Akorn Animal Health) IV or butorphanol (0.02 mg/kg; Torbugesic; Zoetis Inc, Parsippany, NJ, USA) IV for surgical and non-surgical procedures, respectively. Anesthesia was induced with ketamine (2.2 mg/kg; VetaKet; Akorn Animal Health) and midazolam (0.05–0.1 mg/kg; Midazolam injection; Hospira Inc., Lake Forest, IL, USA) IV, and maintained with isoflurane (Akorn Animal Health) in 100% oxygen combined with ketamine (1 mg/kg/hr), xylazine (0.5 mg/kg/hr), and lidocaine (2 mg/kg loading dose followed by 3 mg/kg/hr; VetOne, Boise, ID, USA). Following anesthesia, horses were placed in a recovery stall and received xylazine 0.18–0.55 mg/kg IV to delay anesthetic recovery allowing time to expire isoflurane. Thirty-four horses received 0.004–0.02 mg/kg of acepromazine (VetOne, Boise, ID, USA) either prior to induction of anesthesia or prior to recovery. All horses were recovered on a pad or air mattress, with the aid of head and tail ropes.

### 2.3. Pain scoring

Pain was assessed with both CPS and EQUUS-FAP scoring systems as described elsewhere (4) on the day prior to anesthesia and at 1, 2, 3, 4, and 5 h following anesthetic recovery to standing. All pain scores were performed by 5 veterinary students that received training in regard to the use of both scoring systems. At each timepoint, the assessment was performed independently using both scales by two students simultaneously in a quiet environment. A coin toss was used to randomize the order of the scoring systems at each time point and each horse was scored by the same two students at all time points. Although the students were not masked to the procedure (surgical *vs.* non-surgical), they were unaware of the specific objectives and hypotheses of the study.

The CPS, a simple descriptive scale, requires 5 min to complete and includes physiologic data (physical examination including heart rate, respiratory rate, rectal temperature and digestive sounds), behavioral data (posture, appetite, sweating, kicking at abdomen, pawing at floor, head movements, overall appearance) and response to treatment (interactive behavior, response to palpation). Each variable ranges from 0 to 3 with an overall maximum score of 39. For non-surgical procedures, the scorer palpated the area of concern that was imaged (e.g., joint associated with lameness). The EQUUS-FAP requires 2 min to complete and includes only quiet observation of the patient. Variables assessed are associated with facial expression and each ranged from 0 to 2 with an overall maximum score of 18.

Additional information recorded included signalment, reason for and duration of anesthesia, duration of recovery, anesthetic agents, and perioperative NSAID. No treatments were administered to any patient by any individual involved in the study. Additional analgesic agents were administered at the discretion of the attending Veterinary Teaching Hospital clinician that was not involved with the study. If additional analgesic agents were administered, the horse was subsequently excluded from data collection.

### 2.4. Data analysis

A priori sample size calculation was performed using G*Power 3.1 (Heinrich-Heine-Universität Dusseldorf, Germany). Pain scoring data from a previous clinical study were utilized for the calculation (12). For the CPS, to detect a difference of 1.7 between baseline pain scores and post anesthetic recovery, with an alpha of 5% and power of 95%, a sample size of 40 horses would be required. Therefore, we aimed to observe 50 horses over the course of the study.

Agreement tables with individual veterinary student observers' scores for CPS and EQUUS-FAP were created to calculate Cohen's Kappa. The agreement was defined as: near perfect if 0.80–1.00, substantial if 0.61–0.80, moderate if 0.41–0.60, fair if 0.21–0.40, and none if 0.00–0.20. The individual scores for each timepoint were averaged to determine the overall score for that time point. A generalized linear mixed model with fixed effects of type of procedure (surgical vs. non-surgical), timepoint, and their interaction was used for the analysis. Horse was included as a random effect. The Dunnett's test for multiple comparisons was used for items where a significant effect was found.

## 3. Results

### 3.1. Summary of subjects and procedures

A total of 70 horses met the inclusion criteria with 20 horses excluded due to lack of availability of investigators for post anesthetic assessment, resulting in a total sample size of 50 horses. The study population included: 33 geldings, 14 mares, and 3 stallions of various breeds (Table 1), with a mean ± SD age of 11 ± 6 years and weighing 546 ± 67 kg. Twenty-six horses underwent surgical procedures and 24 non-surgical procedures. Of the former, 21 were orthopedic surgeries (arthroscopy, tenoscopy, neurectomy, dorsal spinous process excision, tooth extraction) and 5 were soft tissue surgeries (wound explore/debride, castration, removal of scirrhous cord, tumor ablation, enucleation). The non-surgical procedures included 22 for magnetic resonance imaging (MRI) and 2 for computed tomography (CT) scans. No complications were noted for any of the anesthetic events and all horses recovered successfully to standing.

**Table 1:** Breeds of horses (*n* = 50) included in the study.

Breed	Number enrolled
Warmblood	20
Quarter horse	11
Irish sport horse	4
Thoroughbred	4
Andalusian	2
Trakehner	2
Friesian	1
Welsh cross	1
Pony	1
Cleveland bay	1
Lusitano	1
Morgan	1
Tennessee walker	1

### 3.2. Pain scores

For the CPS and the EQUUS-FAP, Cohen's Kappa (95% confidence interval) was 0.95 (0.94–0.97) and 0.78 (0.71–0.84), respectively.

**Figure 1:**
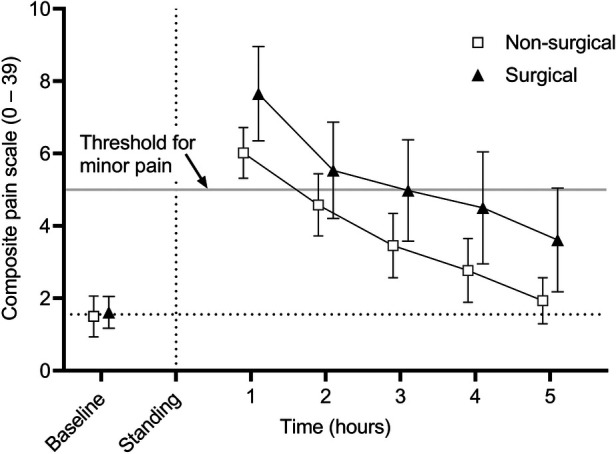
Mean (± 95% confidence interval) composite pain scores (CPS) in 50 adult horses undergoing surgical or non-surgical procedures prior to anesthesia (baseline) and in the hours immediately following recovery to standing. Overall scores are significantly greater than baseline at hours 1-5 following anesthetic recovery (*p* < 0.001). There was no significant effect of reason for anesthesia.

CPS scores are presented in Figure 1. There was no effect of type of procedure (surgical vs. non-surgical) (*p* = 0.6841); however, there was an effect of time (*p* < 0.001), with hours 1–5 being significantly greater than baseline (*p* < 0.001 for all). In regard to individual categories of the scoring system, there was no effect of type of procedure, but there was a significant effect of time (*p* < 0.006 for all). Physiologic scores were significantly greater than baseline at hours 1–5 (*p* < 0.02 for all), behavioral scores were significantly greater than baseline at hours 1–4 (*p* < 0.011 for all), and response to treatment was significantly greater than baseline at hour 1 (*p* = 0.024). There was no interaction between reason for anesthesia and time for any of the analyses.

**Figure 2:**
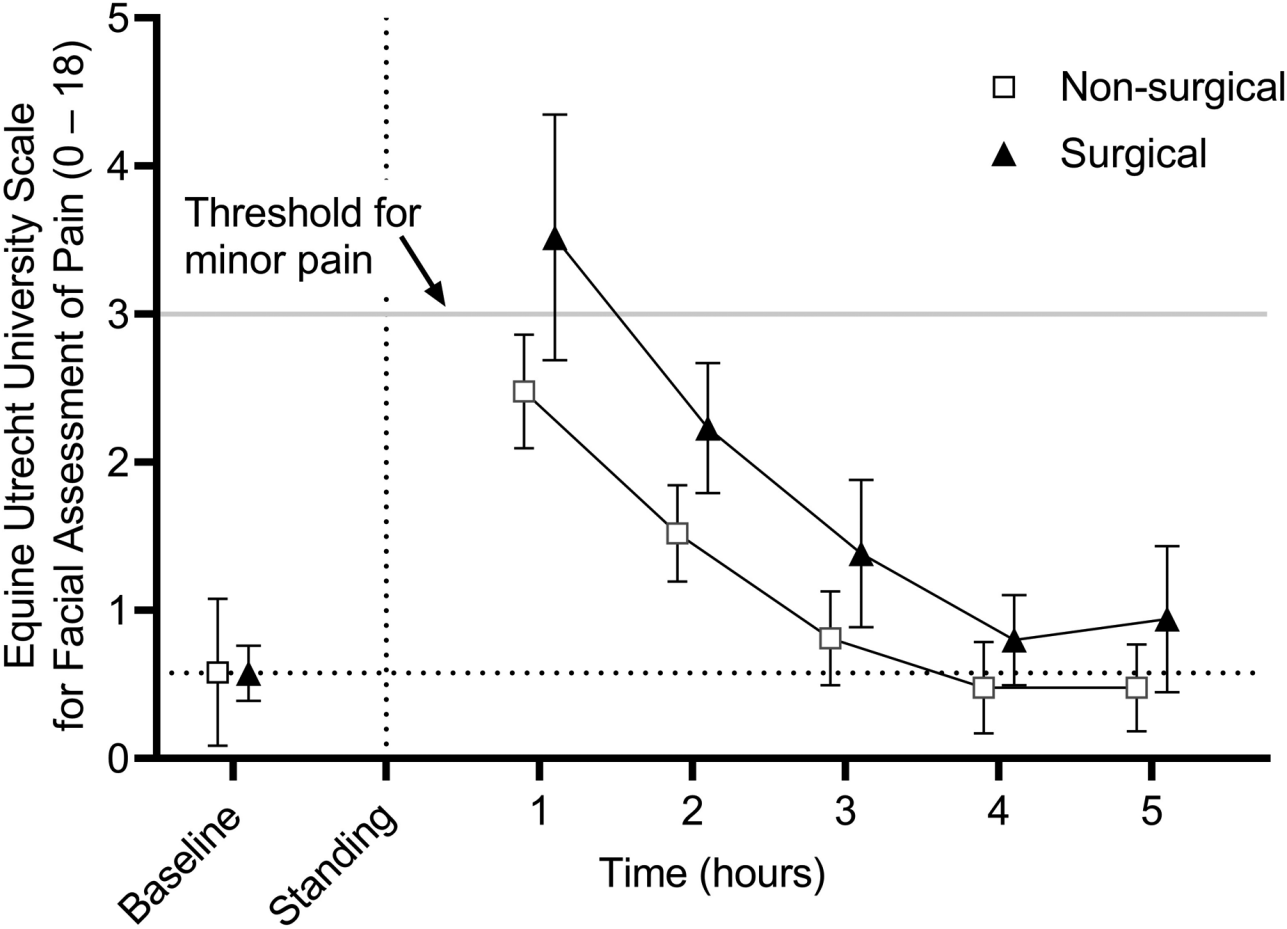
Mean (± 95% confidence interval) equine Utrecht university scale for facial assessment of pain (EQUUS-FAP) scores in 50 adult horses undergoing surgical or non-surgical procedures prior to anesthesia (baseline) and in the hours immediately following recovery to standing. Overall scores are significantly greater than baseline at hours 1 and 2 post anesthesia recovery (*p* < 0.001 for both). There was no significant effect of reason for anesthesia.

EQUUS-FAP scores are presented in Figure 2. There was no effect of type of procedure (surgical vs. non-surgical) (*p* = 0.7942); however, there was a significant effect of time (*p* < 0.001) with hours 1 and 2 being significantly greater than baseline (*p* < 0.001 for both). There was no interaction between reason for anesthesia and time.

There was no effect of anesthetic time or recovery time on CPS (*p* = 0.619 and *p* = 0.2411, respectively) and on EQUUS-FAP (*p* = 0.220, *p* = 0.1821, respectively). No horse received additional analgesic agents within the period of data collection.

## 4. Discussion

In the study presented here, all horses, regardless of reason for anesthesia, were assigned higher CPS and EQUUS-FAP pain scores in the hours immediately following anesthetic recovery to standing in comparison to baseline. In previous studies evaluating the efficacy of these scoring systems in horses undergoing general anesthesia, the first assessment occurred no earlier than 4 h following anesthetic recovery (4, 5, 13). Indeed, van Loon et al. evaluated the role of general anesthesia on CPS after surgical and non-surgical procedures and found no effect. However, in that study, the first pain score was recorded at 4 h post-anesthetic recovery and therefore it is possible that any effect prior to that time point had been missed. Additionally, only 6 horses were included in the non-surgical group and the authors did not report the results of a power analysis, citing small sample size as a limitation to the study.

In a study comparing hydromorphone and butorphanol for analgesia in horses undergoing elective arthroscopy, it was found that horses receiving hydromorphone had CPS scores significantly higher than baseline at 2 h post-anesthetic recovery but returned to baseline at 4 h. Meanwhile, horses receiving butorphanol scored significantly higher than baseline at both 2 and 4 h post anesthetic recovery (12). However, this increase was not observed with the EQUUS-FAP system and the authors speculated that the residual effects of general anesthesia affected the CPS scores at the 2 h timepoint. A similar observation was made in a study comparing buprenorphine and butorphanol for pain management in equine patients undergoing elective surgery. Horses receiving buprenorphine had lower pain scores than butorphanol when using a simple descriptive scale, but this effect did not occur until 3 h following recovery (14). Opioids are known to cause temporary central nervous system excitation immediately following administration in unsedated, non-painful horses (15–17). It is possible that these initial elevated scores were affected by the opioids. However, in the present study the first post-anesthetic pain score was at least 2 h after opioid administration for all horses in the study, and a residual effect of these drugs after this time was unlikely.

The stress response to general anesthesia in horses has been well described with evidence of a substantial adrenocortical response with various anesthetic protocols and patient scenarios (9–11). This stress response may have contributed to the effects on the physiologic measures of the CPS observed here. Partial intravenous anesthesia with ketamine and morphine infusions has been shown to cause increased sympathetic response in comparison to dexmedetomidine infusion with morphine or remifentanil (9). Post-anesthetic cardiopulmonary variables were not monitored in that study, so it is unknown if the enhanced sympathetic tone resulted in altered physiologic response following anesthetic recovery. Therefore, it is possible that augmented sympathetic tone from persisting effects of ketamine in the present study could have contributed to the physiologic and behavioral CPS scores. In cats anesthetized with either alfaxalone or ketamine, post-anesthetic composite pain scale scores were higher in the ketamine group compared to the alfaxalone group (18). In the present study, horses received ketamine for induction of anesthesia and as part of a partial intravenous anesthesia protocol and it is possible that the effect observed may be at least partially attributed to ketamine. However, the horses received several anesthetic agents and it is impossible to determine the role of ketamine in the elevated pain scores observed and further studies are needed to establish this effect.

Difficulty in assessing immediate post-anesthetic pain has also been noted in humans. Ledowski et al. studied the use of a heart rate variability based analgesia nociception index (ANI) as a non-verbal method for post-anesthetic pain assessment in people undergoing non-emergency surgery (19). ANI was compared to the standard self-assessment numeric rating scale (NRS). It was found that ANI was unable to establish different states of acute postoperative pain and the correlation with NRS was deemed weak.

One of the components of pain score validation is construct validity, which is the ability of the test to measure the concept it was designed to evaluate. A component of construct validity is discriminant validity, showing the ability of the scale to measure only the construct it is designed to evaluate and no other constructs that may be existing simultaneously (20). In our study, the residual effects of anesthesia appear to be captured by both the CPS and EQUUS-FAP scales, resulting in elevated pain scores and revealing weakness in the discriminant validity of the scoring systems in the hours immediately following anesthetic recovery.

Equine CPS scores of 5–8 are considered to be consistent with mild pain, 8–10 moderate pain, and >10 severe pain (4). On average, horses included in this study received scores consistent with mild pain at 1 and 2 h before falling below a score of 5. EQUUS-FAP scores of 3–5 are considered consistent with mild pain, 5–8 moderate pain, and >8 is considered severe (4). On average, horses received scores consistent with mild pain at hour 1 following anesthetic recovery before falling below a score of 3. These results suggest that in order to avoid the effects of anesthetic agents on post-anesthetic pain scoring, the first pain scores should be scheduled after 3 h for the CPS and after 2 h for the EQUUS-FAP following anesthetic recovery to standing. With these results in mind, the analgesic plan for each patient should be tailored in accordance with the patient and the procedure, with knowledge that pain scores may not accurately reflect the patient's pain level until the aforementioned time points. Nevertheless, the effects observed here appear to elevate the scores only to the category of mild pain and analgesia should not be withheld from a patient when the clinician believes that it is warranted based on the procedure and clinical signs.

## 5. Limitations

This study has some limitations which should be considered. It is unclear what level of post-anesthetic discomfort may have been present in the non-surgical group. All subjects were endotracheally intubated following placement of a mouth gag, hoisted onto a padded table, maintained under anesthesia, hoisted again onto a mattress in the recovery stall, and pulled by the halter and tail to assist the recovery phase. It is possible that horses may suffer some persistent discomfort following anesthetic recovery associated with these events. Nevertheless, all subjects received an NSAID and an opioid prior to anesthesia, infusions of analgesic agents during maintenance of anesthesia, and an alpha-2 agonist in recovery, which should have provided analgesia during the post-anesthetic period. Additionally, the students performing the pain scoring were not blinded to the type of procedure and this may have affected their scores. However, there was no effect of reason for anesthesia (surgical vs. non-surgical procedure) on the pain scores observed. The surgical procedures that horses in this study underwent did not result in significant pain in the post-operative period with the analgesic agents that were administered. Therefore, a difference between groups may have been detected if the horses had undergone more painful surgeries. Nevertheless, the aim of this study was not to establish if pain was effectively managed but rather to determine if general anesthesia affected pain scores. Lastly, as this was an observational study, the subjects were varied in signalment, presentation, and procedure that was performed which may have affected the pain scores.

## 6. Conclusion

Pain scores as measured by CPS and EQUUS-FAP in equine patients recovering from general anesthesia for surgical and non-surgical procedures are elevated above baseline in the hours immediately following general anesthesia. The results presented here question the validity of these scoring systems in the hours immediately following anesthesia. Further research is indicated to determine if other pain scoring systems may be more useful in pain assessment in the hours immediately following anesthetic recovery.

## Data Availability

The raw data supporting the conclusions of this article will be made available by the authors, without undue reservation.

## References

[1] Bowen IM Redpath A Dugdale A Burford JH Lloyd D Watson T BEVA Primary care clinical guidelines: analgesia. Equine Vet J. (2020) 52(1):13–27. 10.1111/evj.13198 31657050

[2] de Grauw JC van Loon JP. Systematic pain assessment in horses. Vet J. (2016) 209:14–22. 10.1016/j.tvjl.2015.07.030 26831169

[3] van Loon J Van Dierendonck MC. Objective pain assessment in horses (2014–2018). Vet J. (2018) 242:1–7. 10.1016/j.tvjl.2018.10.001 30503538

[4] van Loon J Van Dierendonck MC. Pain assessment in horses after orthopaedic surgery and with orthopaedic trauma. Vet J. (2019) 246:85–91. 10.1016/j.tvjl.2019.02.001 30902195

[5] van Loon JP Jonckheer-Sheehy VS Back W van Weeren PR Hellebrekers LJ. Monitoring equine visceral pain with a composite pain scale score and correlation with survival after emergency gastrointestinal surgery. Vet J. (2014) 200(1):109–15. 10.1016/j.tvjl.2014.01.003 24491373

[6] van Loon JP Van Dierendonck MC. Monitoring acute equine visceral pain with the equine Utrecht university scale for composite pain assessment (EQUUS-COMPASS) and the equine Utrecht university scale for facial assessment of pain (EQUUS-FAP): a scale-construction study. Vet J. (2015) 206(3):356–64. 10.1016/j.tvjl.2015.08.023 26526526

[7] Bussieres G Jacques C Lainay O Beauchamp G Leblond A Cadore JL Development of a composite orthopaedic pain scale in horses. Res Vet Sci. (2008) 85(2):294–306. 10.1016/j.rvsc.2007.10.011 18061637

[8] VanDierendonck MC van Loon JP. Monitoring acute equine visceral pain with the equine Utrecht university scale for composite pain assessment (EQUUS-COMPASS) and the equine Utrecht university scale for facial assessment of pain (EQUUS-FAP): a validation study. Vet J. (2016) 216:175–7. 10.1016/j.tvjl.2016.08.004 27687948

[9] Fujiyama M Jones T Duke-Novakovski T. Evaluation of the perioperative stress response from dexmedetomidine infusion alone, with butorphanol bolus or remifentanil infusion compared with ketamine and morphine infusions in isoflurane-anesthetized horses. Vet Anaesth Analg. (2021) 48(3):344–55. 10.1016/j.vaa.2021.01.006 33741263

[10] Taylor PM. Equine stress responses to anaesthesia. Br J Anaesth. (1989) 63(6):702–9. 10.1093/bja/63.6.702 2692673

[11] Taylor PM. Effects of surgery on endocrine and metabolic responses to anaesthesia in horses and ponies. Res Vet Sci. (1998) 64(2):133–40. 10.1016/S0034-5288(98)90008-X 9625469

[12] Reed R Trenholme N Skrzypczak H Chang K Ishikawa Y Barletta M Comparison of hydromorphone and butorphanol for management of pain in equine patients undergoing elective arthroscopy: a randomized clinical trial. Vet Anaesth Analg. (2022) 49(5):490–8. 10.1016/j.vaa.2022.05.006 35752564

[13] van Loon JPAM Back W Hellebrekers LJ van Weeren PR. Application of a composite pain scale to objectively monitor horses with somatic and visceral pain under hospital conditions. J Equine Vet Sci. (2010) 30(11):641–9. 10.1016/j.jevs.2010.09.011

[14] Taylor PM Hoare HR de Vries A Love EJ Coumbe KM White KL A multicentre, prospective, randomised, blinded clinical trial to compare some perioperative effects of buprenorphine or butorphanol premedication before equine elective general anaesthesia and surgery. Equine Vet J. (2016) 48(4):442–50. 10.1111/evj.12442 25772950 PMC5033022

[15] Combie J Dougherty J Nugent E Tobin T. The pharmacology of narcotic analgesics in the horse. IV. Dose and time response relationships for behavioral responses to morphine, meperidine, pentazocine, anileridine, methadone, and hydromorphone. Eq Med Surg. (1979) 3:377–85.

[16] Hamamoto-Hardman BD Steffey EP McKemie DS Kass PH Knych HK. Meperidine pharmacokinetics and effects on physiologic parameters and thermal threshold following intravenous administration of three doses to horses. BMC Vet Res. (2020) 16(1):368. 10.1186/s12917-020-02564-4 32998730 PMC7528573

[17] Hamamoto-Hardman BD Steffey EP Weiner D McKemie DS Kass P Knych HK. Pharmacokinetics and selected pharmacodynamics of morphine and its active metabolites in horses after intravenous administration of four doses. J Vet Pharmacol Ther. (2019) 42:401–10. 10.1111/jvp.12759 30919469

[18] Buisman M Wagner MC Hasiuk MM Prebble M Law L Pang DS. Effects of ketamine and alfaxalone on application of a feline pain assessment scale. J Feline Med Surg. (2016) 18(8):643–51. 10.1177/1098612X15591590 26088567 PMC10816383

[19] Ledowski T Tiong WS Lee C Wong B Fiori T Parker N. Analgesia nociception index: evaluation as a new parameter for acute postoperative pain. Br J Anaesth. (2013) 111(4):627–9. 10.1093/bja/aet111 23611914

[20] Boateng GO Neilands TB Frongillo EA Melgar-Quiñonez HR Young SL. Best practices for developing and validating scales for health, social, and behavioral research: a primer. Front Public Health. (2018) 6:149. 10.3389/fpubh.2018.00149 29942800 PMC6004510

